# Supplementary material for: Elf1 promotes transcription-coupled repair in yeast by using its C-terminal domain to bind TFIIH
Source: Nat Commun. 2024 Jul 23;15:6223. doi: 10.1038/s41467-024-50539-y (PMC11266705; doi:10.1038/s41467-024-50539-y)
Supplement: Supplementary file 1 — Supplementary Information [file 41467_2024_50539_MOESM1_ESM.pdf]

SUPPLEMENTARY MATERIALS

Supplementary Table 1: Dissociation constants (K_d) for GST-Elf1-CTD and PH domain of p62 interaction measured using Bio-Layer Interferometry (BLI)

PH-p62 (µM)	Kd (µM) repeat1	Kd (µM) repeat2	Kd (µM) repeat3	Mean Kd (µM)	STDEV Kd (µM)
30	2.88	1.70	2.92	2.50	± 0.69
15	5.77	4.89	3.29	4.65	± 1.26
7.5	4.81	4.12	4.54	4.49	± 0.35
3.75	3.80	3.57	3.50	3.62	± 0.16
**SUM**				3.82	± 1.09

Supplementary Table 2: Dissociation constants (K_d) for GST-elf1-Y99A-CTD and PH domain of p62 interaction measured using Bio-Layer Interferometry (BLI)

PH-p62 (µM)	Kd (µM) repeat1	Kd (µM) repeat2	Kd (µM) repeat3	Mean Kd (µM)	STDEV Kd (µM)
30	9.00	7.45	7.31	7.92	± 0.94
15	9.26	8.63	7.59	8.49	± 0.84
7.5	6.77	7.48	7.29	7.18	± 0.37
3.75	5.74	5.46	5.82	5.67	± 0.19
**SUM**				7.32	± 1.24

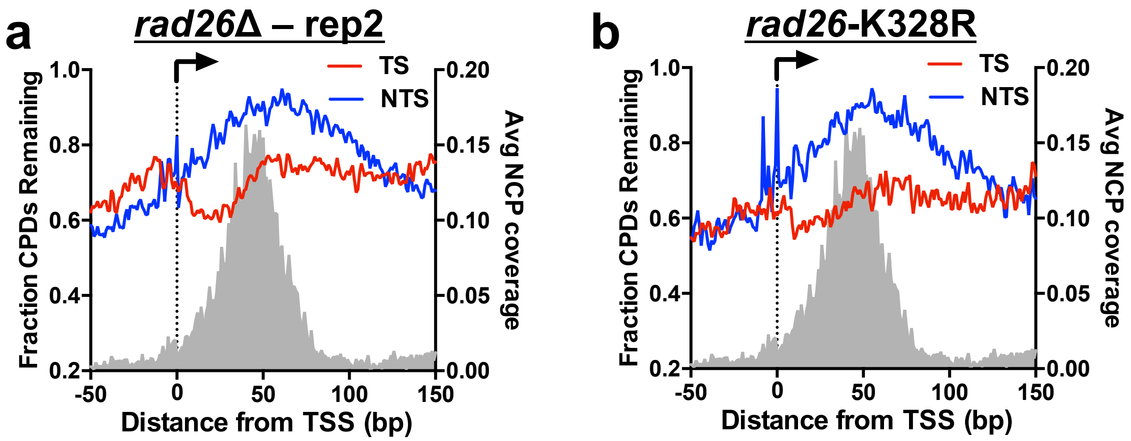

Supplementary Figure 1. Rad26-independent TC-NER immediately downstream of the transcription start site. a, CPD-seq data from *rad26*Δ replicate 2 (data from [1]) and **b,** *rad26*-K328R (catalytically inactive mutant) cells were analyzed immediately adjacent to the transcription start site (TSS) of ~5200 yeast genes on both the transcribed strand (TS) and non-transcribed strand (NTS). Fraction of unrepaired CPDs after 2hr repair relative to 0hr control is depicted. Gray background depicts dyad positions of +1 nucleosome based on [2].

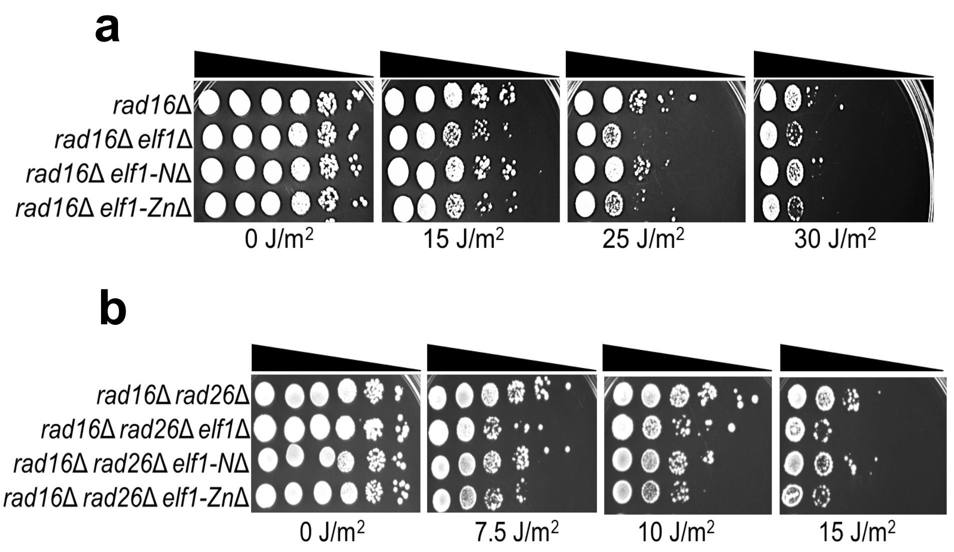

Supplementary Figure 2. The zinc finger domain, but not the N-terminal domain of Elf1 is important for UV survival. a-b, Indicated mutant yeast strains were 10-fold serially diluted, spotted on the YPD plates, and exposed to the indicated doses of UV light. Plates were photographed after 3 days of incubation in the dark.

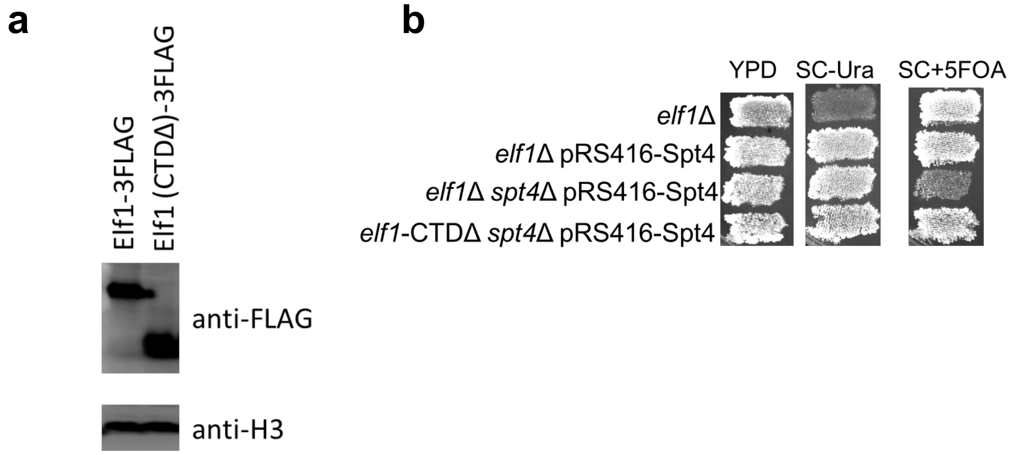

Supplementary Figure 3. Deletion of Elf1 C-terminal domain does not affect protein stability of Elf1 and is not synthetic lethal with *SPT4*. a, Western blot analysis using anti-FLAG or anti-histone H3 (loading control) was used to probe total protein extracts isolated from the indicated yeast strains. **b,** Unlike *elf1*Δ, *elf1*-CTDΔ is not synthetic lethal with *spt4*Δ. Indicated mutant yeast strains were tested for their growth on different media as shown. SC+5-FOA media selects against the presence of pRS416-Spt4 plasmid, resulting in a *spt4*Δ strain.

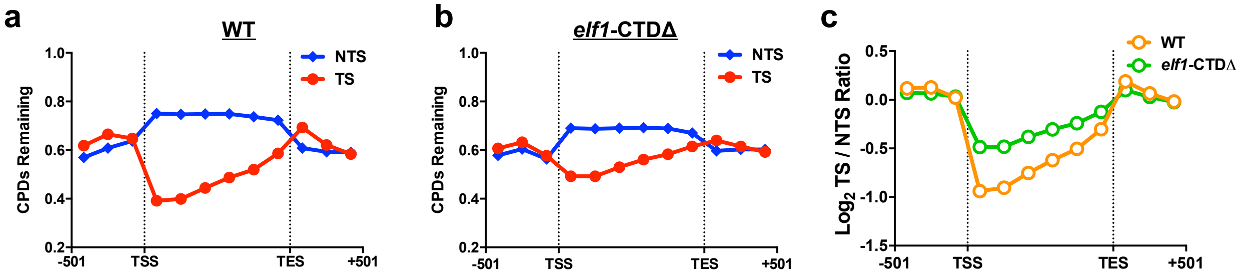

Supplementary Figure 4. Elf1 C-terminal domain is critical for TC-NER. a-b, The fraction of CPDs remaining after 2 hr of repair relative to 0hr was plotted for both **a** WT (data from [3]) and **b** *elf1*-CTDΔ along the transcribed strand (TS) and non-transcribed strand (NTS) for ~5000 yeast genes. CPD-seq data for each gene was divided into 6 equally sized bins and the fraction of CPDs remaining was analyzed in each bin. The fraction of CPDs remaining was also analyzed for 3 bins each consisting of 167 bp of flanking DNA upstream of transcription start site (TSS) or downstream of transcription end site (TES) for each gene. **c,** The $\log_2$ ratio of unrepaired CPDs on the TS relative to the NTS in the WT and *elf1*-CTDΔ cells was plotted to quantify the TC-NER defect in *elf1*-CTDΔ cells. Less efficient repair of the TS by TC-NER in *elf1*-CTDΔ cells is indicated by the $\log_2$ TS/NTS being closer to zero.

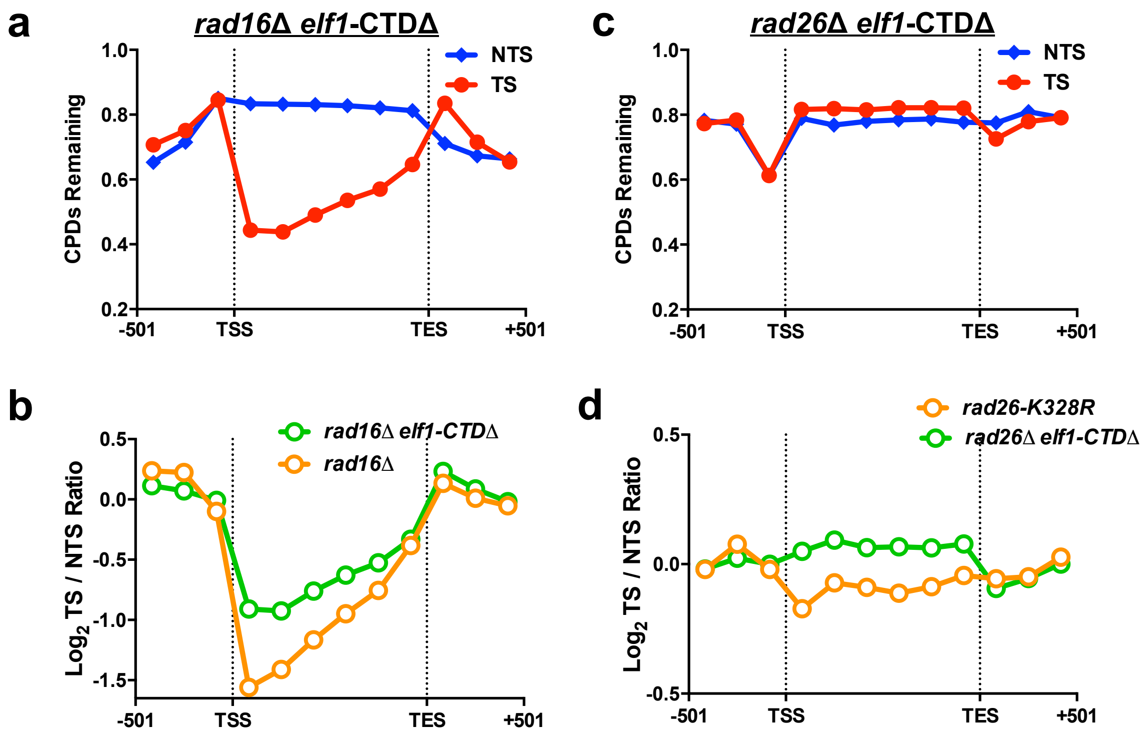

Supplementary Figure 5. C-terminal domain of Elf1 is critical for Rad26-independent TC-NER. a, The average fraction of CPDs remaining after 2 hr of repair relative to 0hr control was normalized to the damage counts (0hr) was plotted for both the transcribed strand (TS) and non-transcribed strand (NTS) for ~5000 yeast genes, as described in Supplementary Fig. 4 above. CPD-seq data for *rad16*Δ *elf1*-CTDΔ is depicted. **b**, Log2 TS/NTS ratio of unrepaired CPDs after 2hr repair for ~5000 yeast genes for *rad16*Δ *elf1*-CTDΔ cells relative to *rad16*Δ control from Bohm et al., 2021[4]. **c**, Same as panel **a**, except CPD-seq data for *rad26*Δ *elf1*-CTDΔ cells is depicted. **d**, Same as panel **d**, except comparing data from *rad26*Δ *elf1*-CTDΔ cells with *rad26*-K328R control (data from [1]).

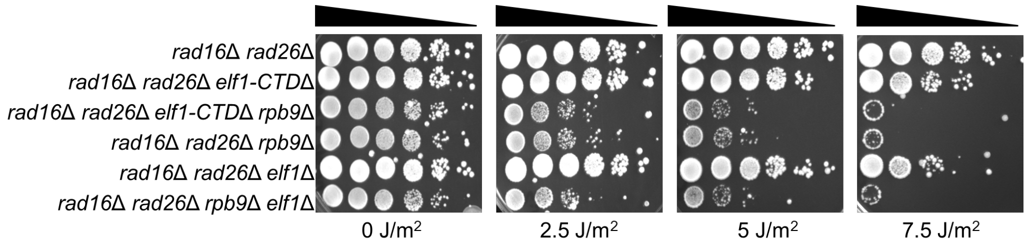

Supplementary Figure 6. Residual Rad26-independent TC-NER in *elf1*-CTDΔ (or *elf1*Δ) mutant is dependent on Rpb9. Indicated mutant yeast strains were 10-fold serially diluted, spotted on the YPD plates, and exposed to the indicated doses of UV light. Plates were photographed after 3 days of incubation in the dark.

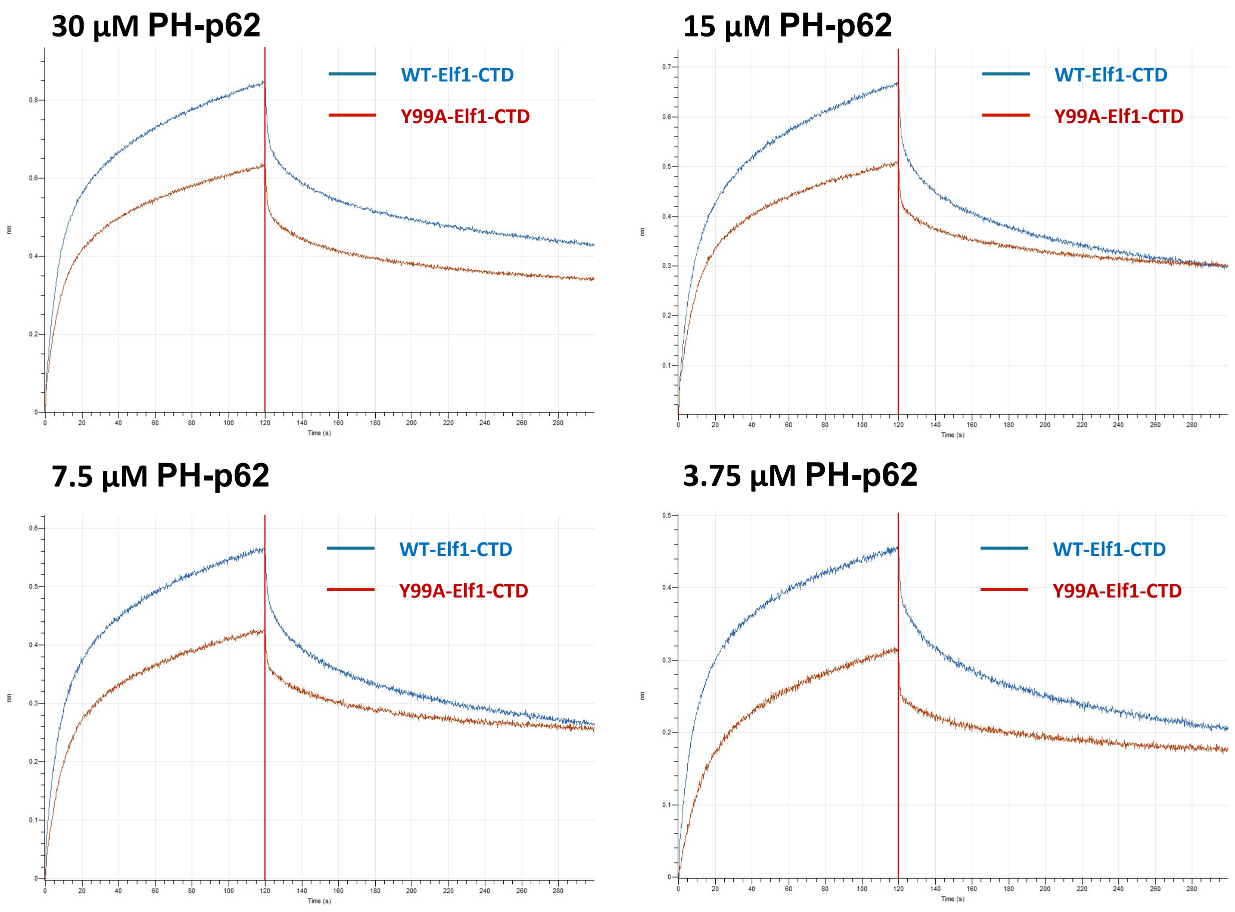

Supplementary Figure 7. Representative BLI traces for GST-Elf1-CTD interactions with the PH Domain of p62 at varying concentrations. Traces were obtained from Biolayer Interferometry (BLI) to examine the interactions between the PH domain of p62 and two variants of GST-Elf1-CTD: the Wild Type (WT, in blue) and the Y99A mutant (in red). Each graph delineates the binding kinetics at specific PH-p62 concentrations, ranging from 30 µM to 3.75 µM. The kinetics profiles illustrate a 2-minute association phase with the PH-p62 and a subsequent 3-minute dissociation phase in BLI buffer, which were critical in determining and comparing the K_d values for each Elf1-CTD variant.

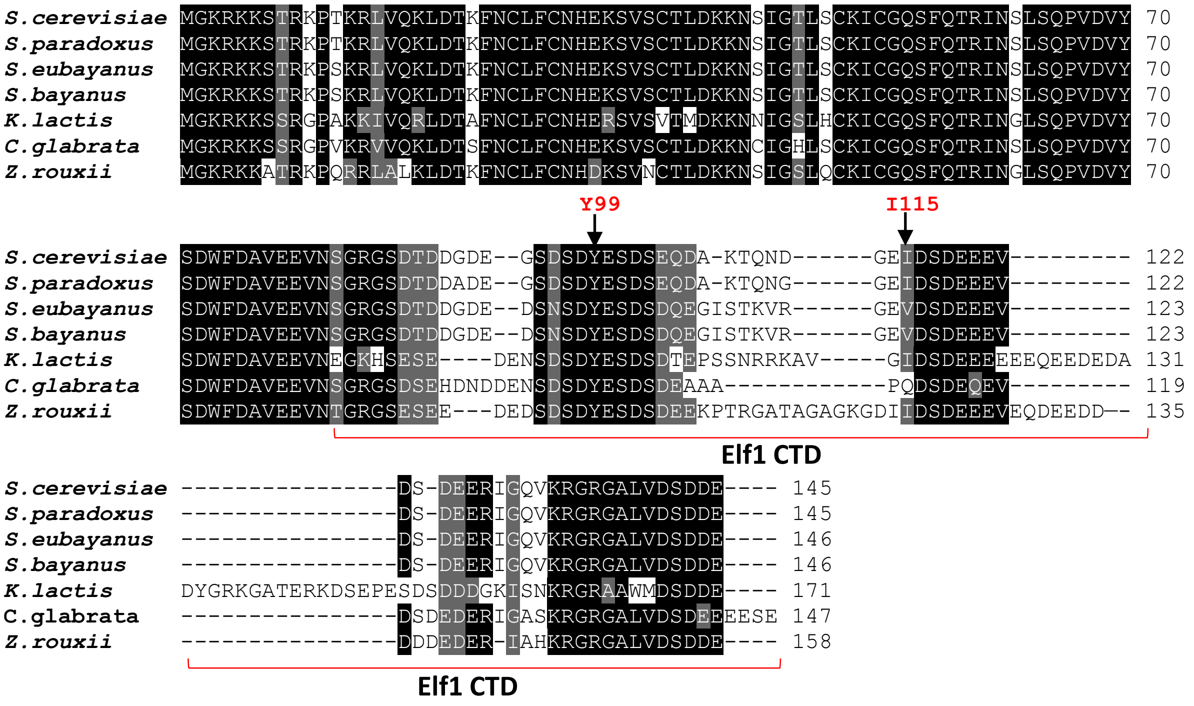

Supplementary Figure 8. Sequence alignment of Elf1 protein sequences from different yeasts. Alignment of Elf1 protein sequences from different yeasts as indicated. The aromatic amino acid, Y99 in *S. cerevisiae* Elf1 C-terminal domain (Elf1-CTD), is conserved across different yeasts.

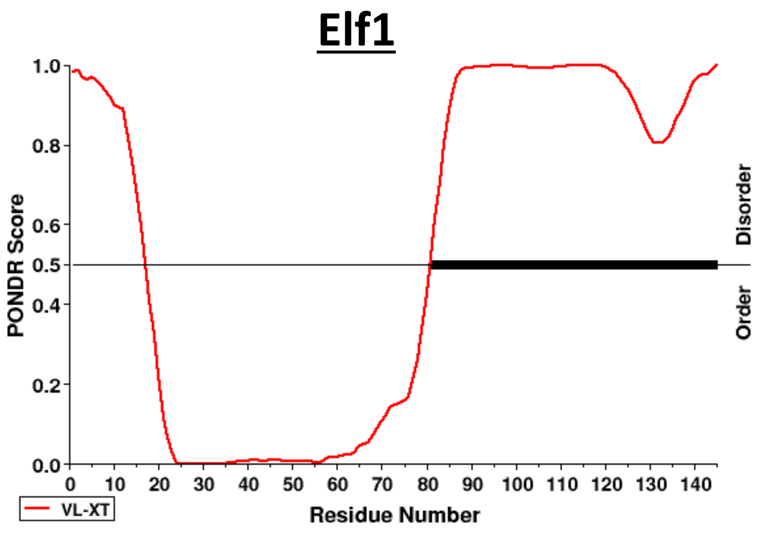

Supplementary Figure 9. Elf1 CTD is intrinsically disordered. Analysis of Elf1 protein sequence using PONDR software[5] revealed the presence of a predicted intrinsically disordered region in the Elf1 CTD (residues 85 to 145).

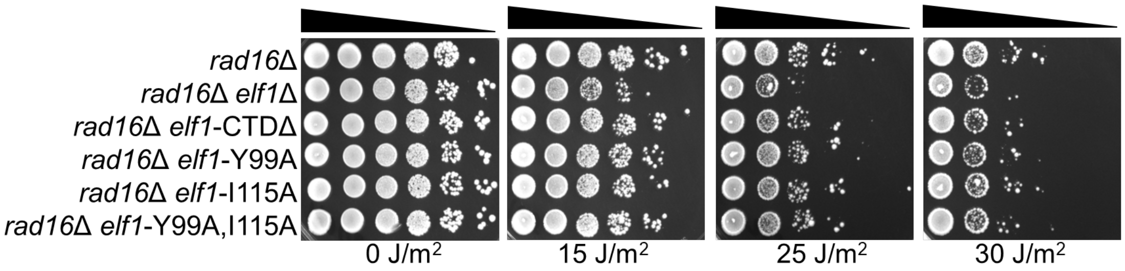

Supplementary Figure 10. Elf1 Y99 is important for UV survival. Indicated mutant yeast strains were 10-fold serially diluted, spotted on the YPD plates, and exposed to the indicated doses of UV light. Plates were photographed after 3 days of incubation in the dark.

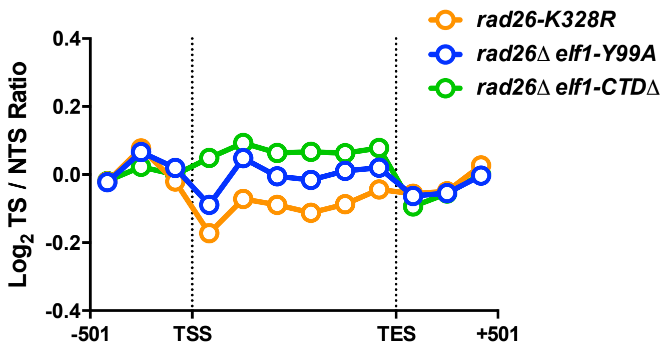

Supplementary Figure 11. Plot of Log_2 TS/NTS ratio for unrepaired CPDs after 2hr relative to 0hr control for ~5000 yeast genes for the indicated yeast strains. Data for *rad26*-K328R control from [1].

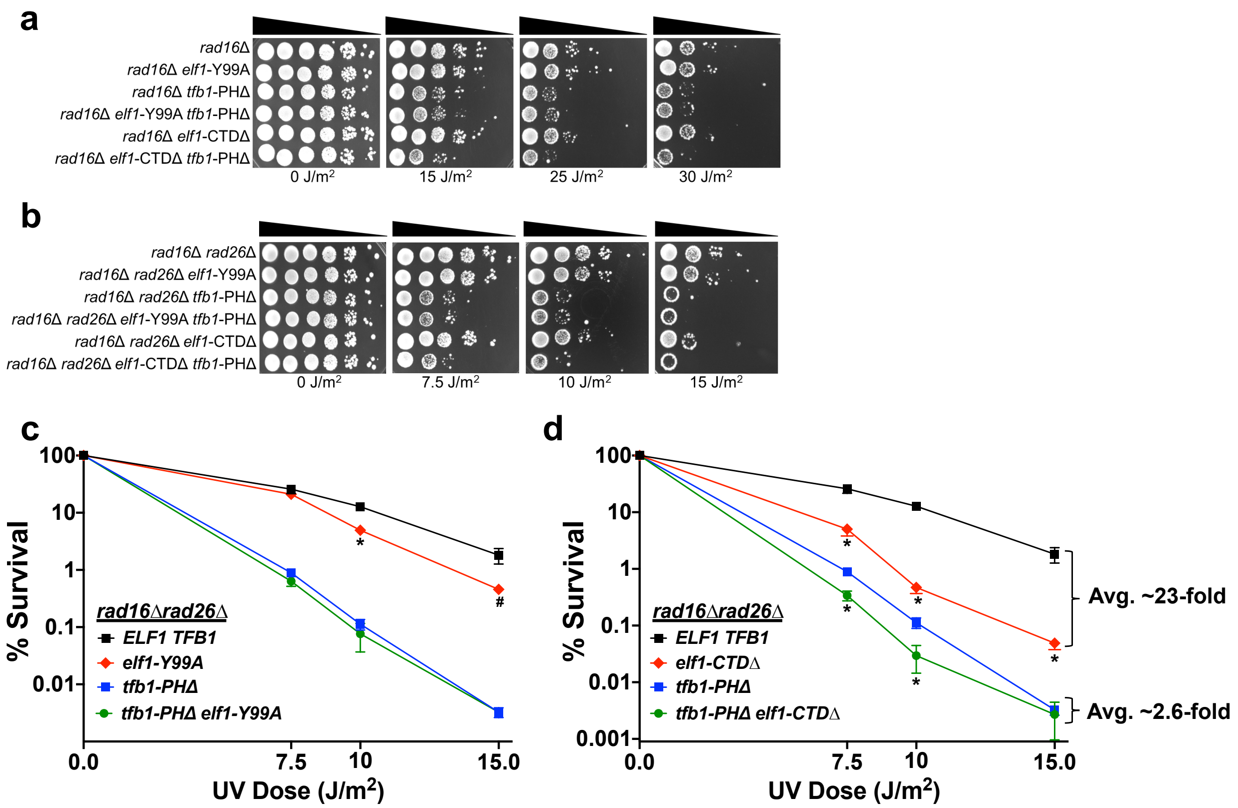

Supplementary Figure 12. UV sensitivity of *elf1*-Y99A mutant is epistatic with deletion of the PH domain of the p62 subunit (i.e., *tfb1*-PHΔ) of TFIIH. a-b, Indicated mutant yeast strains were 10-fold serially diluted, spotted on the YPD plates, and exposed to the indicated doses of UV light. Plates were photographed after 3 days of incubation in the dark. The UV sensitivity of the *elf1*-CTDΔ also appears to be largely epistatic with the *tfb1*-PHΔ mutant. **c-d**, Quantitative UV sensitivity data measured at the specified UV doses and for the indicated yeast mutants in a *rad16*Δ *rad26*Δ mutant background. The *ELF1 TFB1* and *tfb1*-PHΔ data are the same for both graphs, and included for comparison. Mean ± SEM is depicted for n = 4 replicates. *P < 0.05 based on two-sided *t*-test with Holm-Sidak correction for multiple hypothesis testing. [#]P = 0.049, but adjusted P-value with Holm-Sidak correction is 0.095.

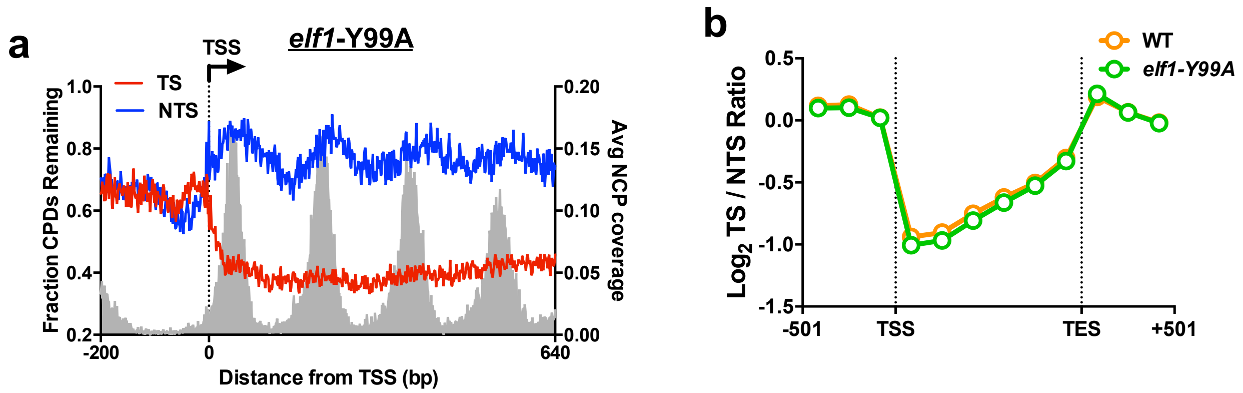

Supplementary Figure 13. Elf1 Y99 is not required for Rad26-dependent TC-NER.

a, Analysis of CPD-seq data from *elf1*-Y99A cells for ~5,200 genes at single nucleotide resolution on both the transcribed strand (TS) and non-transcribed strand (NTS). The fraction of unrepaired CPDs after 2hr of repair (relative to 0hr) is depicted. The gray peaks (right *y* axis) depict average nucleosome coverage for nucleosome dyads from MNase nucleosome map[2]. **b,** Log_2 ratio of unrepaired CPDs on the TS relative to the NTS in the WT and *elf1*-Y99A cells.

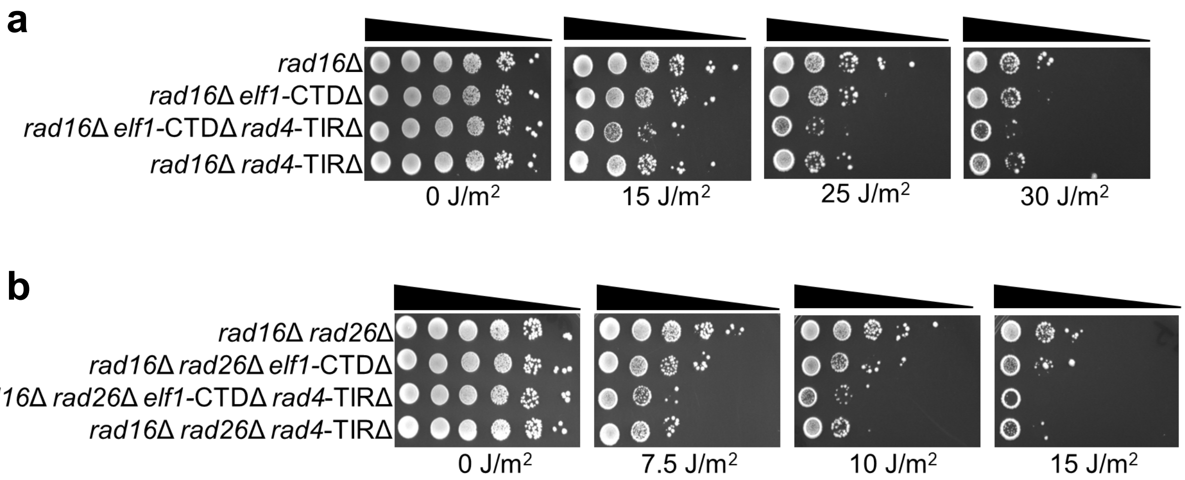

Supplementary Figure 14. Simultaneous mutation of Elf1-CTD and Rad4-TIR shows enhanced UV sensitivity in *rad16*Δ and *rad16*Δ *rad26*Δ backgrounds. a-b, Indicated mutant yeast strains were 10-fold serially diluted, spotted on YPD plates, and exposed to different doses of UV light. Plates were photographed after ~3 days of incubation in the dark

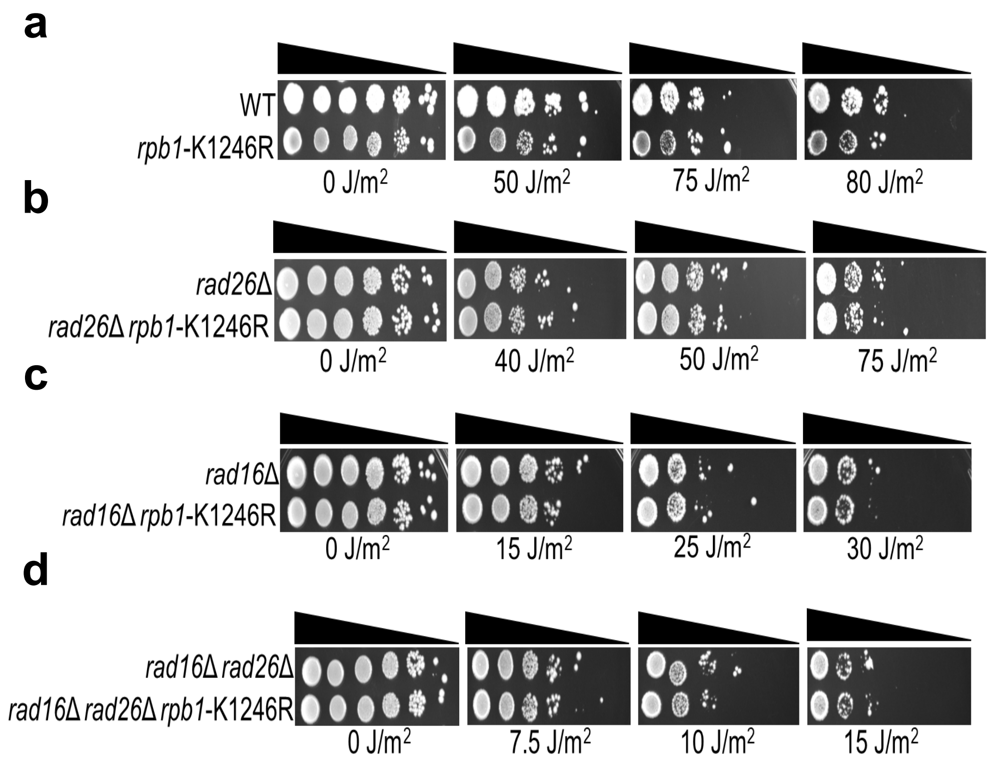

Supplementary Figure 15. The putative Rpb1 ubiquitylation site at K1246 is not important for UV survival in yeast. a-d, Indicated mutant yeast strains were 10-fold serially diluted, spotted on YPD plates, and exposed to different doses of UV light. Plates were photographed after 3 days of incubation in the dark.

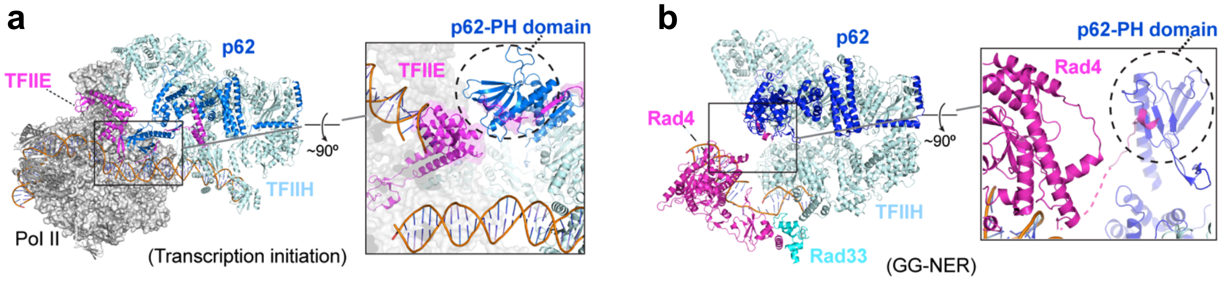

Supplementary Figure 16. Structures showing TFIIH recruitment in transcription initiation and GG-NER. a, During transcription initiation, TFIIH is recruited through the interaction between TFIIE and the PH domain of p62 (PDB ID: 5OQJ). **b,** During GG-NER, TFIIH is recruited through the interaction between Rad4 (XPC) and the PH domain of p62 (PDB ID: 7K04).

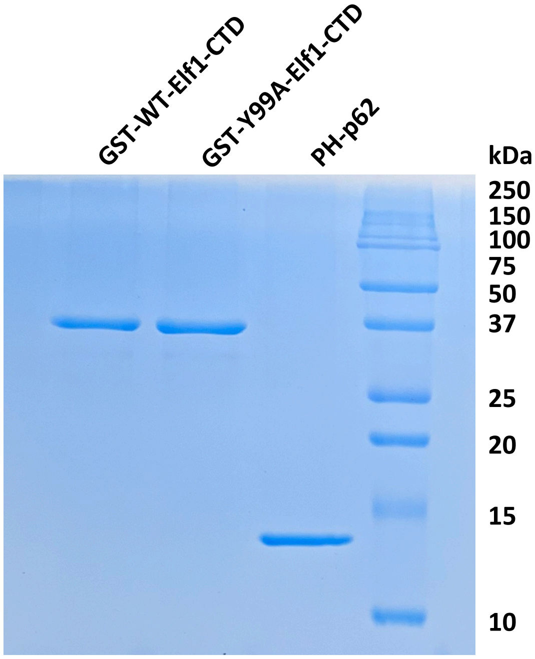

Supplementary Figure 17: SDS-PAGE analysis of purified proteins used in BLI Assays. Lanes 1 to 3 display the Coomassie Blue-stained proteins: GST-tagged Wild Type Elf1-CTD (GST-WT-Elf1-CTD), GST-tagged Y99A mutant Elf1-CTD (GST-Y99A-Elf1-CTD), and the PH domain of p62 (PH-p62). Far right lane is molecular weight markers (sizes in kDa are indicated).

Supplementary References

1. Duan M, Selvam K, Wyrick JJ, Mao P. Genome-wide role of Rad26 in promoting transcription-coupled nucleotide excision repair in yeast chromatin. *Proceedings of the National Academy of Sciences of the United States of America* **117**, 18608-18616 (2020).

2. Weiner A*, et al.* High-resolution chromatin dynamics during a yeast stress response. *Molecular cell* **58**, 371-386 (2015).

3. Geijer ME*, et al.* Elongation factor ELOF1 drives transcription-coupled repair and prevents genome instability. *Nature Cell Biology* **23**, 608-619 (2021).

4. Bohm KA*, et al.* Distinct roles for RSC and SWI/SNF chromatin remodelers in genomic excision repair. *Genome research* **31**, 1047-1059 (2021).

5. Obradovic Z, Peng K, Vucetic S, Radivojac P, Dunker AK. Exploiting heterogeneous sequence properties improves prediction of protein disorder. *Proteins* **61 Suppl 7**, 176-182 (2005).
