# Supplementary material for: Elf1 promotes transcription-coupled repair in yeast by using its C-terminal domain to bind TFIIH
Source: Nat Commun. 2024 Jul 23;15:6223. doi: 10.1038/s41467-024-50539-y (PMC11266705; doi:10.1038/s41467-024-50539-y)
Supplement: Supplementary file 3 — Description of Additional Supplementary Files [file 41467_2024_50539_MOESM3_ESM.pdf]

Description of Additional Supplementary Files

File Name: Supplementary Data 1
Description: Sequences of oligonucleotides used for yeast gene knockout/CRISPR genome editing and CPD-seq.
