# Supplementary material for: Elf1 promotes transcription-coupled repair in yeast by using its C-terminal domain to bind TFIIH
Source: Nat Commun. 2024 Jul 23;15:6223. doi: 10.1038/s41467-024-50539-y (PMC11266705; doi:10.1038/s41467-024-50539-y)
Supplement: Supplementary file 5 — Reporting Summary [file 41467_2024_50539_MOESM5_ESM.pdf]

nature portfolio

Corresponding author(s): John Wyrick and Dong Wang

Last updated by author(s): Jun 13, 2024

Reporting Summary

Nature Portfolio wishes to improve the reproducibility of the work that we publish. This form provides structure for consistency and transparency in reporting. For further information on Nature Portfolio policies, see our Editorial Policies and the Editorial Policy Checklist.

Statistics

For all statistical analyses, confirm that the following items are present in the figure legend, table legend, main text, or Methods section.

n/a	Confirmed	
☐	☒	The exact sample size (*n*) for each experimental group/condition, given as a discrete number and unit of measurement
☐	☒	A statement on whether measurements were taken from distinct samples or whether the same sample was measured repeatedly
☐	☒	The statistical test(s) used AND whether they are one- or two-sided<br>*Only common tests should be described solely by name; describe more complex techniques in the Methods section.*
☒	☐	A description of all covariates tested
☒	☐	A description of any assumptions or corrections, such as tests of normality and adjustment for multiple comparisons
☐	☒	A full description of the statistical parameters including central tendency (e.g. means) or other basic estimates (e.g. regression coefficient) AND variation (e.g. standard deviation) or associated estimates of uncertainty (e.g. confidence intervals)
☐	☒	For null hypothesis testing, the test statistic (e.g. F, t, r) with confidence intervals, effect sizes, degrees of freedom and P value noted<br>*Give P values as exact values whenever suitable.*
☒	☐	For Bayesian analysis, information on the choice of priors and Markov chain Monte Carlo settings
☒	☐	For hierarchical and complex designs, identification of the appropriate level for tests and full reporting of outcomes
☒	☐	Estimates of effect sizes (e.g. Cohen's d, Pearson's r), indicating how they were calculated

Our web collection on statistics for biologists contains articles on many of the points above.

Software and code

Policy information about availability of computer code

Data collection	None
Data analysis	Bowtie2, SAMtools (version 1.10), BEDTools (version 2.27.1), Java TreeView (version 1.2.0), and custom Perl scripts were used to analyze CPD-seq data. ImageQuant 5.2, Excel and Graphpad Prism (version 6 and 8) were used to analyze alkaline gel electrophresis data. Data from CPD-seq and quantitative UV survival were analyzed using Excel and Graphpad Prism (version 6 and 8). The custom Perl scripts are available at: https://github.com/bmorledge-hampton19/ELF1_CTD . For BLI analysis, Octet system's software (Data Acquisition v12.0.2.11 and Data Analysis HT v12.0.2.59 from FORTEBIO was used.

For manuscripts utilizing custom algorithms or software that are central to the research but not yet described in published literature, software must be made available to editors and reviewers. We strongly encourage code deposition in a community repository (e.g. GitHub). See the Nature Portfolio guidelines for submitting code & software for further information.

Data

Policy information about availability of data

All manuscripts must include a data availability statement. This statement should provide the following information, where applicable:

- Accession codes, unique identifiers, or web links for publicly available datasets
- A description of any restrictions on data availability
- For clinical datasets or third party data, please ensure that the statement adheres to our policy

The CPD-seq data generated in this manuscript have been deposited in the Gene Expression Omnibus (GEO) database under accession code GSE243603 [https://www.ncbi.nlm.nih.gov/geo/query/acc.cgi?acc=GSE243603]. Source data are provided with this paper.
The published CPD-seq data for WT, rad26Δ, and rad16Δ is available from GEO under accession numbers:
GSE161930 [https://www.ncbi.nlm.nih.gov/geo/query/acc.cgi?acc=GSE161930]
GSE145911 [https://www.ncbi.nlm.nih.gov/geo/query/acc.cgi?acc=GSE145911]
GSE149082 [https://www.ncbi.nlm.nih.gov/geo/query/acc.cgi?acc=GSE149082]
GSE131101 [https://www.ncbi.nlm.nih.gov/geo/query/acc.cgi?acc=GSE131101]

Research involving human participants, their data, or biological material

Policy information about studies with human participants or human data. See also policy information about sex, gender (identity/presentation), and sexual orientation and race, ethnicity and racism.

Reporting on sex and gender	Not Applicable
Reporting on race, ethnicity, or other socially relevant groupings	Not Applicable
Population characteristics	Not Applicable
Recruitment	Not Applicable
Ethics oversight	Not Applicable

Note that full information on the approval of the study protocol must also be provided in the manuscript.

Field-specific reporting

Please select the one below that is the best fit for your research. If you are not sure, read the appropriate sections before making your selection.

☒ Life sciences ☐ Behavioural & social sciences ☐ Ecological, evolutionary & environmental sciences

For a reference copy of the document with all sections, see nature.com/documents/nr-reporting-summary-flat.pdf

Life sciences study design

All studies must disclose on these points even when the disclosure is negative.

Sample size	Sample size for CPD-seq and alkaline gel analysis were based on our previously experience (PMID: 27457959 and PMID: 30473425, respectively).
Data exclusions	Initial pilot alkaline gel analysis replicates with experimental or technical issues were excluded from analysis.
Replication	The alkaline gel electrophoresis was replicated three times. Quantitative UV survival assays were replicated three times (Fig. 5c) or four times (Supplementary Fig. 12c,d). The pulldown assay and Biolayer Interferometry (BLI) assay was repeated three times (for BLI, three times for each different protein concentration, for a total of 12 times). All the replicates in each of these experiments were successful.
Randomization	Randomization was not necessary due to using appropriate controls, etc.
Blinding	Blinding was not necessary,as appropriate controls and analysis methods were used

Reporting for specific materials, systems and methods

We require information from authors about some types of materials, experimental systems and methods used in many studies. Here, indicate whether each material, system or method listed is relevant to your study. If you are not sure if a list item applies to your research, read the appropriate section before selecting a response.

Materials & experimental systems

n/a	Involved in the study
	☒ Antibodies
	☒ Eukaryotic cell lines
☒	Palaeontology and archaeology
☒	Animals and other organisms
☒	Clinical data
☒	Dual use research of concern
☒	Plants

Methods

n/a	Involved in the study
☒	ChIP-seq
☒	Flow cytometry
☒	MRI-based neuroimaging

Antibodies

Antibodies used

anti-H3 (Abcam, ab46765)
anti-FLAG (Sigma-Aldrich, F1804)

Validation

anti-H3 (Abcam, ab46765), validated/used in many previous papers (e.g., PMID: 35263330, PMID: 33436623, etc.)
anti-FLAG (Sigma-Aldrich, F1804), validated by manufacturer.

Eukaryotic cell lines

Policy information about cell lines and Sex and Gender in Research

Cell line source(s): Yeast (Saccharomyces cerevisiae) cells

Authentication: Standard yeast genetics methods

Mycoplasma contamination: N/A

Commonly misidentified lines (See ICLAC register): None

Plants

Seed stocks: Not Applicable

Novel plant genotypes: Not Applicable

Authentication: Not Applicable
