# Supplementary figures and images for: Elf1 promotes transcription-coupled repair in yeast by using its C-terminal domain to bind TFIIH
Source: Nat Commun. 2024 Jul 23;15:6223. doi: 10.1038/s41467-024-50539-y (PMC11266705; doi:10.1038/s41467-024-50539-y)

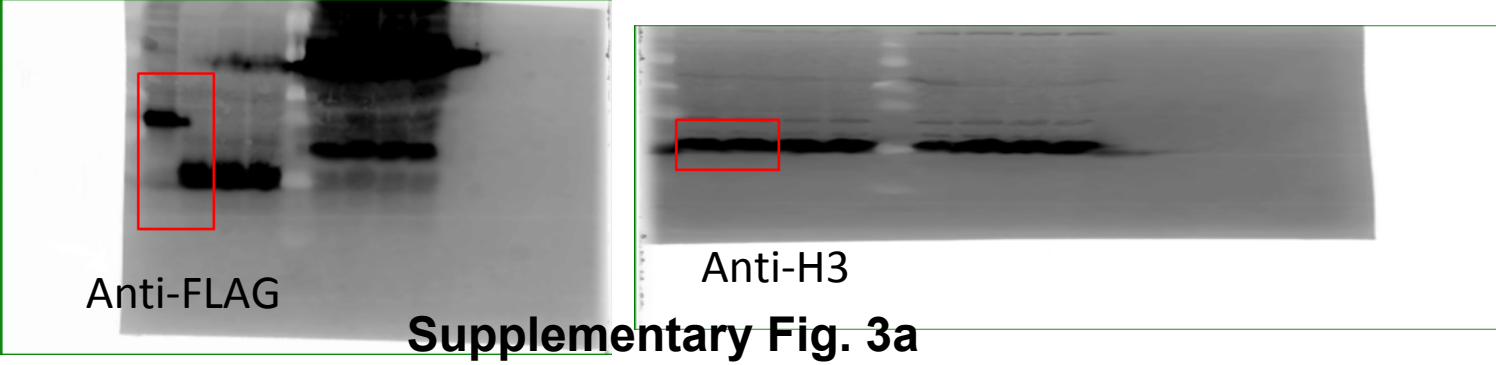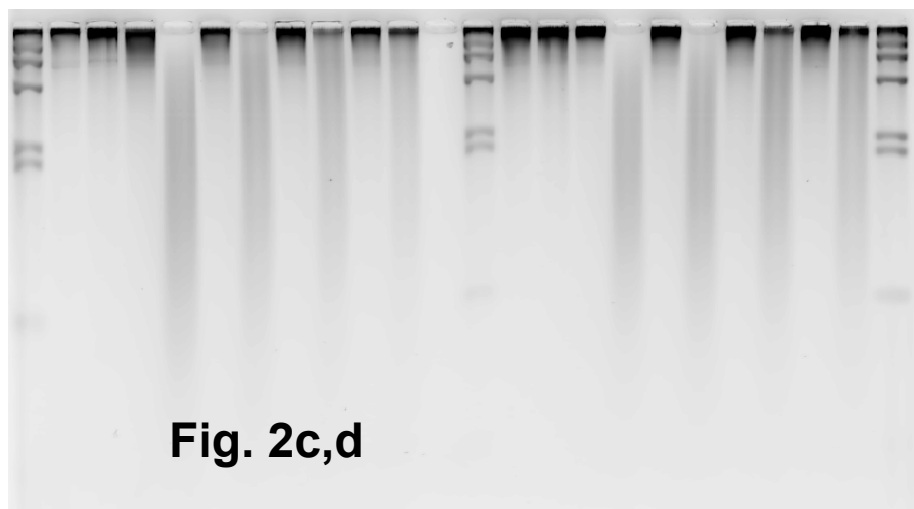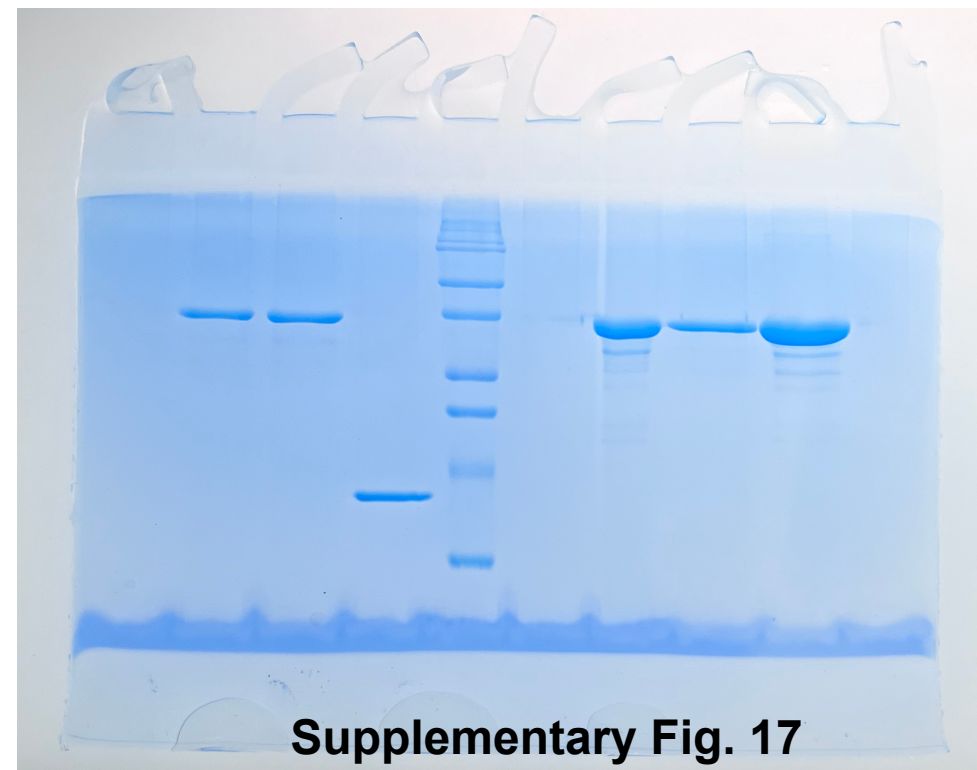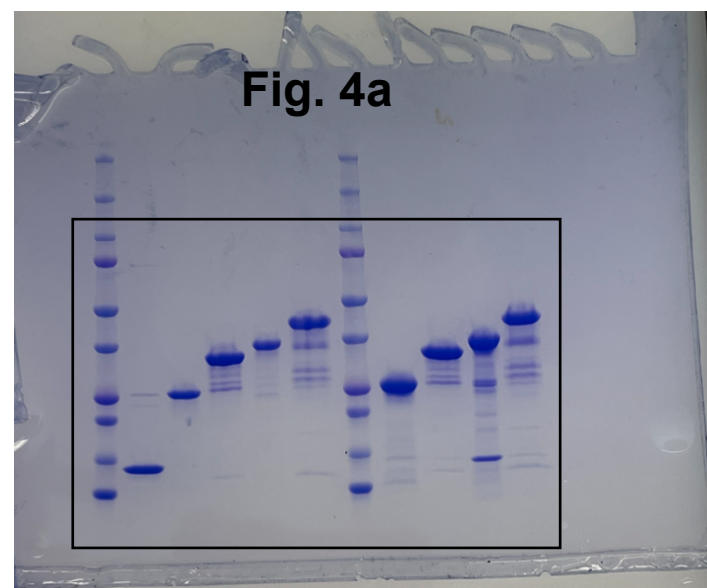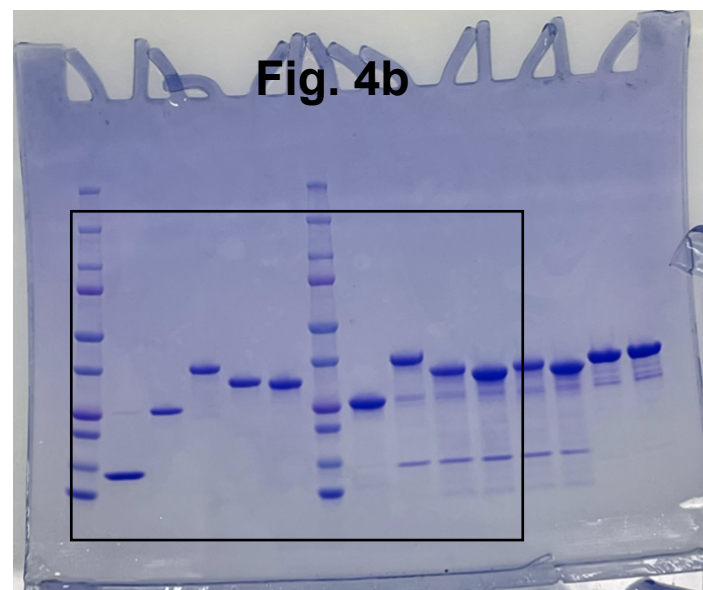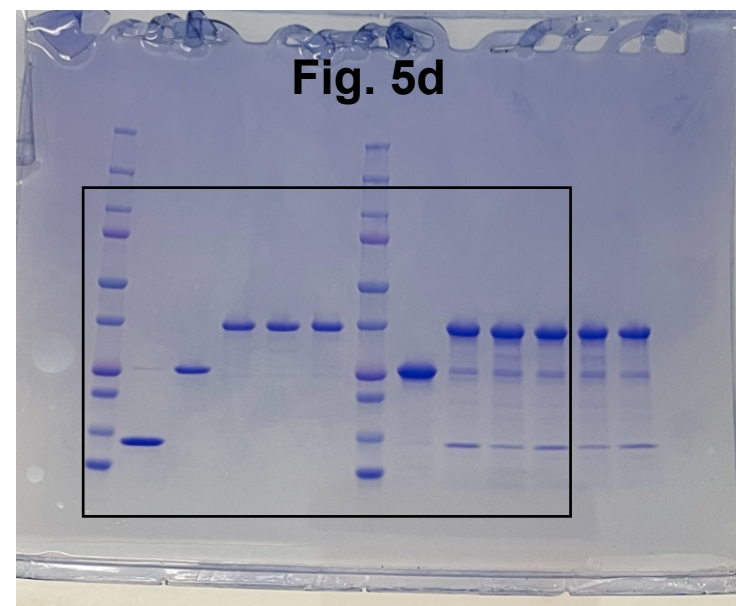

Supplement: Supplementary file 6 — Source Data [file 41467_2024_50539_MOESM6_ESM.zip › SourceDataFiles/UncroppedImages_Blots.pdf]
